# Cardiac angiogenesis directed by stable Hypoxia Inducible Factor-1

**DOI:** 10.1186/2045-824X-5-15

**Published:** 2013-08-29

**Authors:** Chad B Walton, Jennifer Ecker, Cynthia D Anderson, Joel T Outten, Randall Z Allison, Ralph V Shohet

**Affiliations:** 1 Department of Medicine, University of Hawaii, Honolulu, HI 96813, USA; 2 Department of Cellular and Molecular Biology, University of Hawaii, Honolulu, HI 96813, USA

**Keywords:** HIF-1, Angiogenesis, Perfusion, Cardiac

## Abstract

**Background:**

The heterodimeric, oxygen-sensitive transcription factor Hypoxia Inducible Factor-1 (HIF-1) orchestrates angiogenesis and plays a key role in the response to ischemia and the growth of cancers.

**Methods:**

We developed a transgenic mouse line in which expression of an oxygen-stable HIF-1α construct was controlled by a tetracycline-responsive promoter. HIF-1α expression was induced for up to 28 days in adult mouse heart, resulting in angiogenesis and progressive ventricular dysfunction.

**Results:**

Gross inspection demonstrated enlarged hearts with large epicardial vessels with prominent side branches. Perfusion curves obtained by ultrasound contrast analysis demonstrated a significant increase in the myocardial red cell volume after 28 days of HIF-1α expression. Corrosion casts of cardiac vessels were made with a new low-viscosity resin that can fill the vasculature down to the level of the capillaries. Scanning electron microscopy of these casts reveal "lakes" of capillaries forming off of larger vessels after HIF expression, and support the rapid formation of mature neovascularization. Pro-angiogenic factors DLL-4, Notch-1, and PDGF-β, were evaluated by immunohistochemistry and Western blots, and support a pattern of progressive functional neoangiogenesis.

**Conclusions:**

This study demonstrates the structural characteristics of HIF-directed angiogenesis and supports the utility of manipulation of HIF signaling to enhance perfusion and treat ischemia.

## Background

HIF-1 is a heterodimeric transcription factor with α and β subunits that regulate a wide range of angiogenic, metabolic and oxygen-transport related genes [1]. While HIF-1β is a constitutively expressed protein, HIF-1α is rapidly degraded in normoxic conditions [2]. In hypoxic conditions, HIF-1α is stable and can bind to HIF-1β [2] and translocate to the nucleus where the dimer binds to the hypoxia response element (HRE) in HIF-1 regulated genes [3]. In normoxic conditions, two proline residues of HIF-1α, (Pro402 and Pro564) [4], are hydroxylated. These sites are recognized by the von-Hippel Lindau tumor suppressor (pVHL) [5], which directs HIF to proteasomal degradation. Another oxygen-dependent control acting to limit the activity of HIF-1α involves hydroxylation of an asparagine residue (Asn803). This prevents the HIF-1α C-terminal activation domain (CAD) from interacting with the transcriptional co-activating protein p300 [6] thereby reducing the transcriptional activity of the complex [7].

To test the effects of transgenic expression of HIF-1 in the heart of an otherwise normal adult animal, we have created a cardiac-specific, Tet-inducible, oxygen-stabilized transgenic line wherein the induced HIF-1α transgene has been substituted with alanine at both prolines responsible for degradation as well as the asparagine needed for interaction with p300. We have shown that this transgene encodes a stabilized form of HIF-1α that accumulates in normoxic tissue. Here we have gone on to assess the vascular effects of this expression and demonstrate the morphometry and signaling pathways of HIF-regulated neoangiogenesis in the adult mammalian heart.

## Methods

### Animal model

All animal work was approved by the University of Hawaii Institutional Animal Care and Use Committee, (approval number 06-011-4) and utilized 6 to 8 week old male mice. The description of the Tet-regulated HIF-1α-PPN-HA transgenic strain, which used alleles derived from both FVB and C57 strains, is described elsewhere [8]. Double transgenic mice (tTA/HIF-1α-PPN-HA), containing an oxygen-stable mutagenized HA tagged, HIF-1α expression construct under Tet-off control, were maintained on 200 μg doxycycline per ml of 2.5% sucrose-water until HIF-1α-PPN-HA expression was desired. All animals were treated with doxycycline from conception to 6 weeks. Thereafter, doxycycline was omitted for varying periods to assess effects of the mutated HIF-1α transgene.

### Histological staining

Mice (n=6 for each experimental time-point) were euthanized *via* CO_2_ asphyxiation, and then perfused with PBS by cardiac puncture followed by fixation with 15 mL of 4% paraformaldehyde in PBS. For paraffin-embedded tissue, hearts were removed and immersed in 4% paraformaldehyde for 4 hours, then transferred to 70% ethanol. Following dehydration through ethanol and transfer to xylene, hearts were embedded in hot paraffin wax, and five μm sections were cut with a microtome. Prior to staining, slides were deparaffinized in xylene and rehydrated in an ethanol series.

To perform hematoxylin & eosin staining, slides were incubated in Gill number 2 hematoxylin (ThermoScientific, Rockford, IL) for 3 minutes, rinsed under running tap water, then counterstained with eosin Y (EMD Millipore, Billerica, MA) for one minute. After one minute in acetic water (0.1% glacial acetic acid in water), slides were dehydrated in an ethanol series, cleared in xylenes, and coverslips were affixed with Permount (ThermoFisher Scientific, Waltham, MA).

Immunofluorescence experiments were performed on sections from hearts obtained from HIF-1α-PPN-HA uninduced and induced animals at various timepoints after intracardial perfusion with PBS and 4% PFA. Hearts were embedded in Optimal Cutting Temperature (OCT) compound and 10 μm sections were subjected to immunofluorescence labeling using a mouse monoclonal primary antibody to the HA-tag of the transgene (Roche, Branchburg, NJ), and subsequent AlexFluor®-568 (Invitrogen, Carlsbad, CA) secondary antibodies. Immunofluorescence images were obtained using a Zeiss Axioscop 2 Plus microscope with AxioVision Release 4.7 software (Carl Zeiss MicroImaging, LLC, Thornwood, NY). Negative control sections were treated identically but did not include the primary antibody.

### Western blots

Mice were euthanized by CO_2_ asphyxiation. Hearts were rapidly excised, washed in cold PBS, and flash frozen in liquid nitrogen. Individual hearts were then homogenized in RIPA-buffer with Complete Protease Inhibitor cocktail (Roche, Branchburg, NJ), and cleared by centrifugation (1,000 × g at 4°C). Western blots were performed with antibodies directed towards the following; HA-tag of the transgene (Roche, Branchburg, NJ), HIF-1α (Sigma-Aldrich, St Louis, MO), PDGF-β, DLL-4, Notch-1(all gifts from Ed Dratz), and GAPDH (Sigma-Aldrich, St Louis, MO), and secondary AlexFluor®-568-conjugated antibodies (Invitrogen, Carlsbad, CA). Blots were visualized with a Typhoon scanner (GE Lifesciences, Piscataway, NJ) (535 nm excitation, 560LP emission, 500PMT). For quantification, Western blot images were analyzed with the software ImageJ (NIH). All blots were analyzed as the mean densitometry (n=4 biological replicates).

### Corrosion casting

Following transgene induction for one, three, seven, fourteen or twenty-eight days, mice were selected for corrosion casting based on observed cardiac dilation and enlarged epicardial vessels. Double transgenic mice maintained on doxycycline were used as controls as well as tTA+ alone. PU4ii resin was used to cast the hearts according to manufacturer’s protocol (VasQTec, Switzerland) [9]. Briefly, mice were anesthetized with an intraperotineal (IP) injection of Ketamine (150 mg/kg) and Xylazine (20 mg/kg) (both Butler Schien Animal Health, Dublin, OH). The left ventricle was then perfused with 20 ml artificial cerebrospinal fluid (NaCl, 130 mM; KCl, 3.5 mM; KH_2_PO_4_, 1.25 mM; MgSO_4_, 1.5 mM; CaCl_2_, 2.0 mM; NaHCO_3_, 24 mM and glucose, 10 mM), containing Heparin; 25,000 U/L; all Sigma-Aldrich, St Louis, MO), followed by 20 ml of 4% formalin in PBS. Perfusion conditions were a flow rate of 4 ml/min, pressure of 110 mm Hg and temperature of 35°C. The PU4ii resin (VasQtec, Switzerland) was then infused at the same rate and temperature. After allowing the resin to cure for 1–2 days at room temperature, the hearts were macerated in 7.5% KOH, followed by decalcification with 5% formic acid, each for 24 h at 50°C. Casts were washed with water and allowed to air dry for 4 days. The dried casts were gold/palladium sputter-coated for 30 seconds (Hummer® 6.2, Annatech USA, Union City, CA) and viewed with a Hitachi S-800 Field Emission Scanning Electron Microscope with digital image capture using Soft Imaging System analySIS image acquisition (Biological Electron Microscopy Facility at the University of Hawaii). Experimental controls included varying the injection pressures from 80 mm Hg to 200 mm Hg, as well as the resin concentration from 90% to 70% (diluted with 2-butanone).

### Vascular leakage

Vascular leakage was evaluated by perfusion with Evans Blue as described by Udaka et al. [10]. The absorbance of the formamide-eluted dye was measured with a spectrophotometer (Beckman DU530, Beckman, Fullerton, CA) at 620 nm, and the concentration of dye in the tissue was calculated as OD_620_/(50 mg tissue weight). Statistical significance in heart weight to body weight ratios from On Dox animals (n=6) compared to Off Dox 10 days animals (n=6) was done using a Student’s *t* test for paired comparisons using Prism 6 software (GraphPad Software, La Jolla, CA).

### Perfusion curve analysis

Microbubbles were prepared in the laboratory according to published methods [11]. Prior to microbubble delivery, mice were anesthetized by IP injection of Ketamine (150 mg/kg) and Xylazine (20 mg/kg). A high frequency (35 MHz) ultrasound transducer was placed in a stationary position on the thorax of the mouse in a long-axis view using the VEVO 2100 Imaging System (VisualSonics, Montreal, Canada). A video loop of the left ventricle (LV) was obtained, to serve as a baseline for contrast imaging. Microbubbles (1 × 10^6^) were administered intravenously as a bolus through a tail vein catheter while a second video loop of the LV was obtained. A reference set of images was defined from this contrast loop and compared to the baseline study loop. The VEVO 2100 software was used to analyze the difference in intensities, and perform curve analyses (including Change if intensity), based on calculations detailed by Wei et al. [12]. The resultant contrast difference between the two video loops was pseudo-colored for visualization. Non-filtered mean amplitude (linear a.u.) was plotted against time with a threshold of 8% (level which eliminates data that is not relevant to the contrast image), contrast Dynamic Range (DR) equivalent to 10 db (a measure of the intensity level of the overlay image), and normalized to the selected area of interested (anterior LV). Statistical analyses of the perfusion curve change of intensity from “on dox” to “28 off doxycycline” was conducted using a paired student’s *t* test (GraphPad Software, La Jolla, CA).

### Statistical Analysis

All values are presented as mean ± SD. Statistical significance was evaluated with Student’s *t* test for paired comparisons using Prism 6 software (GraphPad Software, La Jolla, CA).

## Results

### Morphology and histology of hearts after HIF-1α-PPN induction

**Figure 1:**
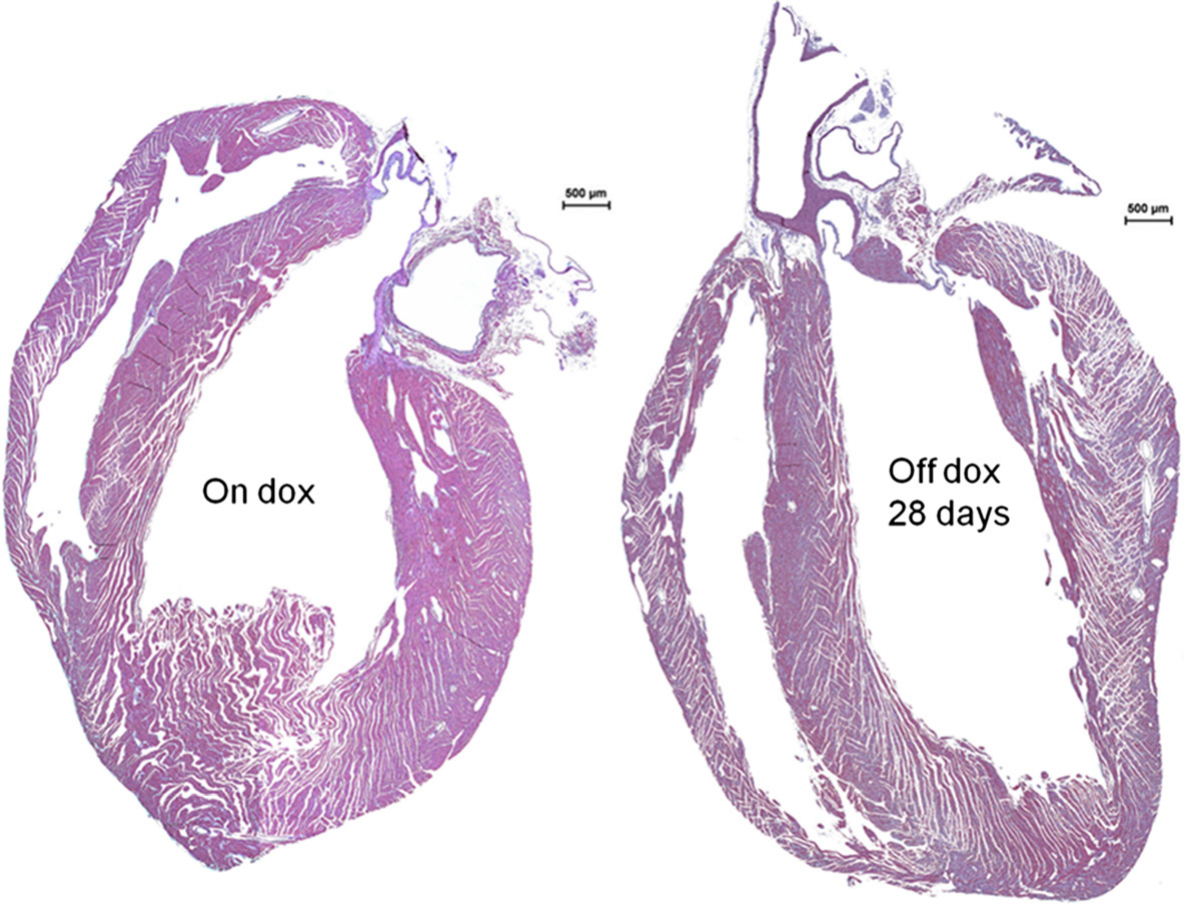
**Cardiac hypertrophy following transgene expression in cardiomyocytes.** Hematoxylin & Eosin staining of hearts from 8 week old mice with the Tet-regulated transgene uninduced (On dox) or induced for four weeks (Off dox 28 days) reveals overt hypertrophy with continuous stable HIF-1α-PPN-HA expression.

Hearts were larger in the tTA/HIF-1α-PPN-HA than in the control tTA+ mice after omitting doxycycline for 28 days, and we have described elsewhere the quantification of dilation and ventricular dysfunction that follows HIF induction [8]. On initial inspection, remarkably large epicardial vessels with prominent side branches were observed [8]. The myocardium showed hypertrophy and heterogeneity of myocyte size (Figure 1).

### Corrosion casts and vascular leakage

**Figure 2:**
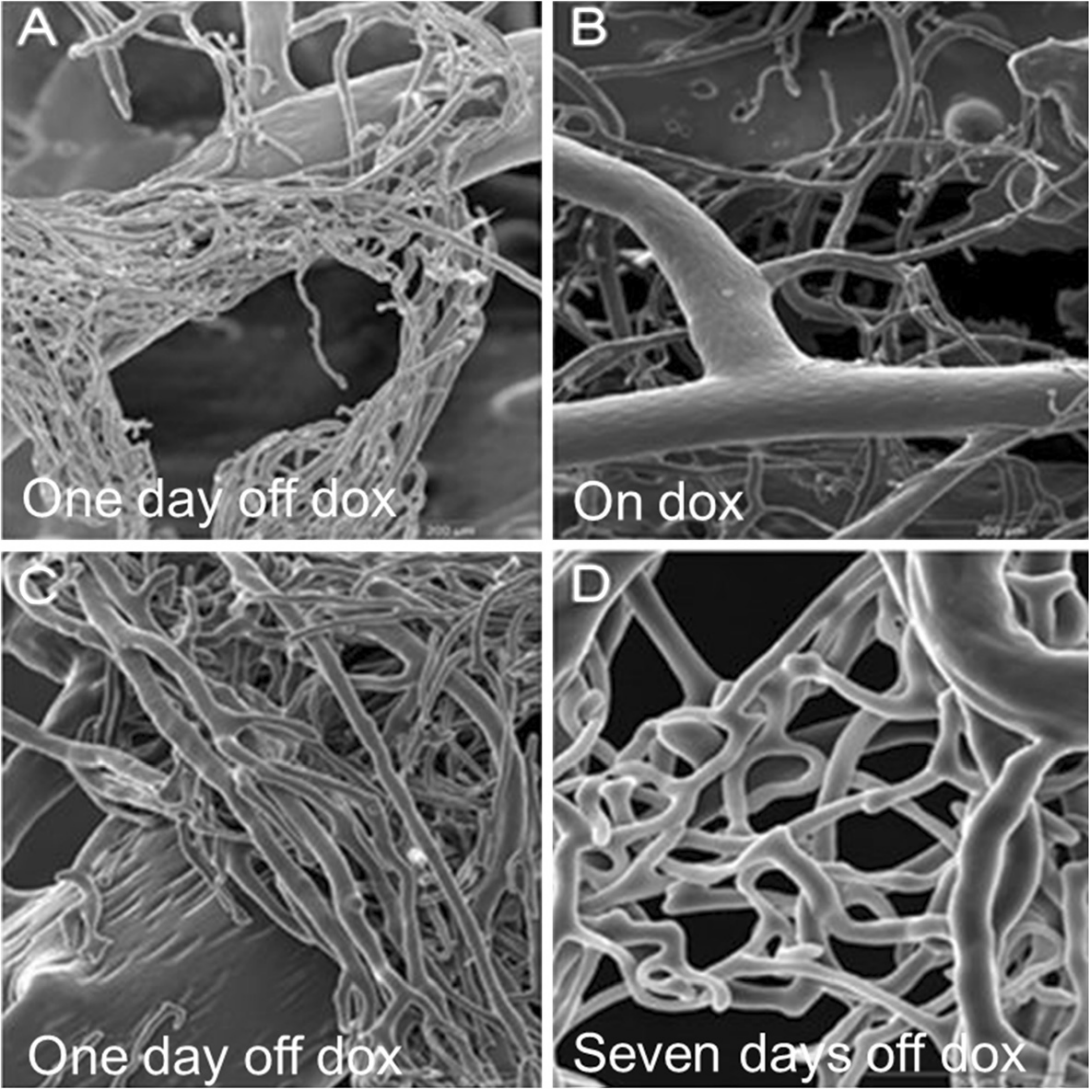
**Scanning electron micrographs of corrosion casts of coronary vasculature.** Eight week old mice were induced to express the stable transgene for one or seven days, or left uninduced. **A**. and **C**. One day after doxycycline removal (One day off dox), **B**. Tet-regulated HIF-1α-PPN-HA mice maintained on doxycycline (On Dox), and **D**. seven days off doxycycline (Seven days off dox). The coronary vessels exhibit rapid neoangiogensis with functional capillary beds by day seven of stable transgene induction. All SEM images focus on coronary vessels.

**Figure 3:**
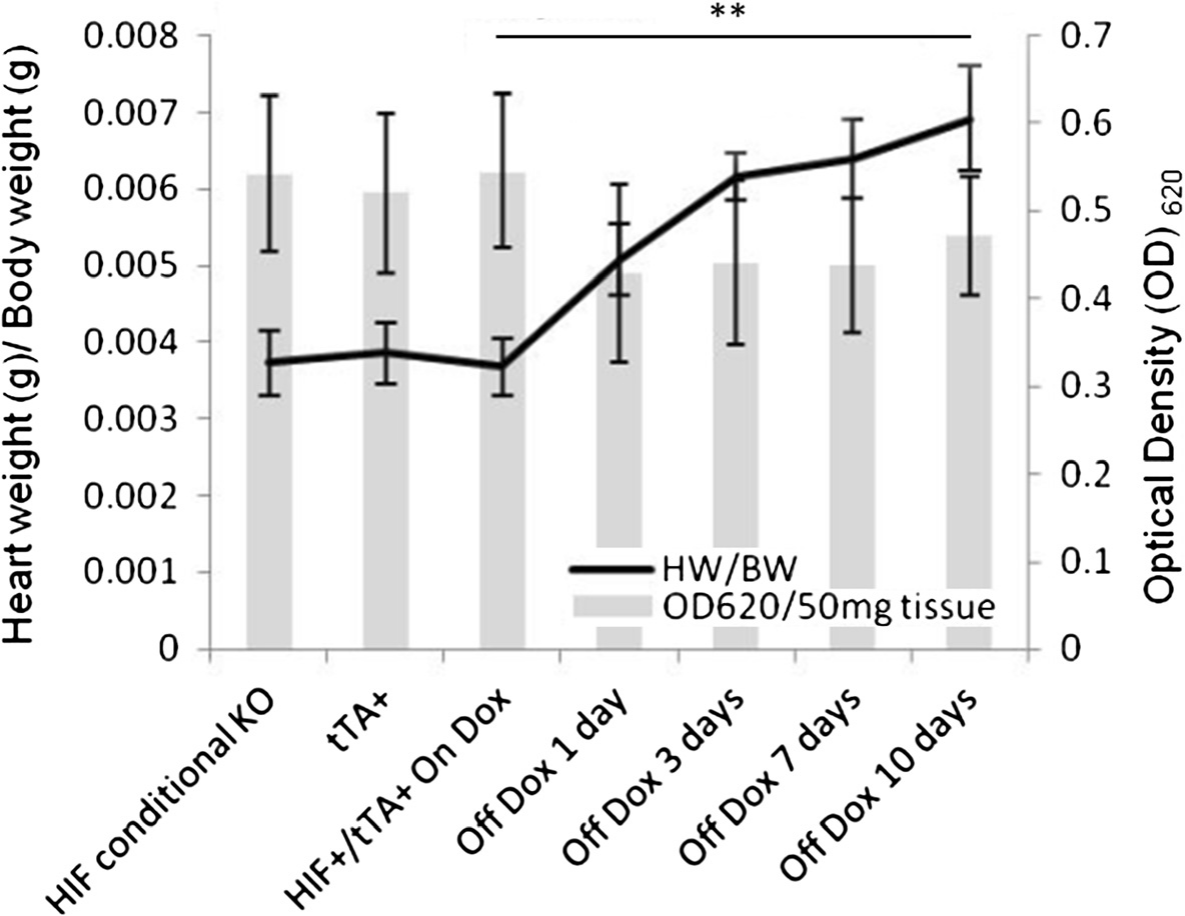
**Myocardial vascular leakage following stable transgene induction for up to ten days.** Vascular leakage was evaluated by perfusion with Evans Blue dye *via* left ventricular injection, and subsequent dye extravasation quantified by absorbance analysis. Even with an increased heart weight (HW) to body weight (BW) ratio, there is no increase in dye release, suggesting the newly formed capillary sized vessels are indeed functional. (n=6 for each analysis). (** denotes p<0.01).

PU4ii corrosion casting of coronary vasculature demonstrated a number of differences between the induced and non-induced mice. After one day of transgene expression (Figure 2A and C), webs of increased capillary density are seen at branch points of coronary vessels; these are not seen in animals maintained on doxycycline (Figure 2B). Seven days off doxycycline; webs of atypical capillary proliferation are seen at some distance from the feeding vessel (Figure 2D), a trend we see continuing through Day 28 (not shown). Additionally, distinct from published reports of VEGF-mediated angiogenesis [13], we do not observe vascular leakage after transgene induction for up to 10 days (Figure 3).

### Perfusion curve analysis

**Figure 4:**
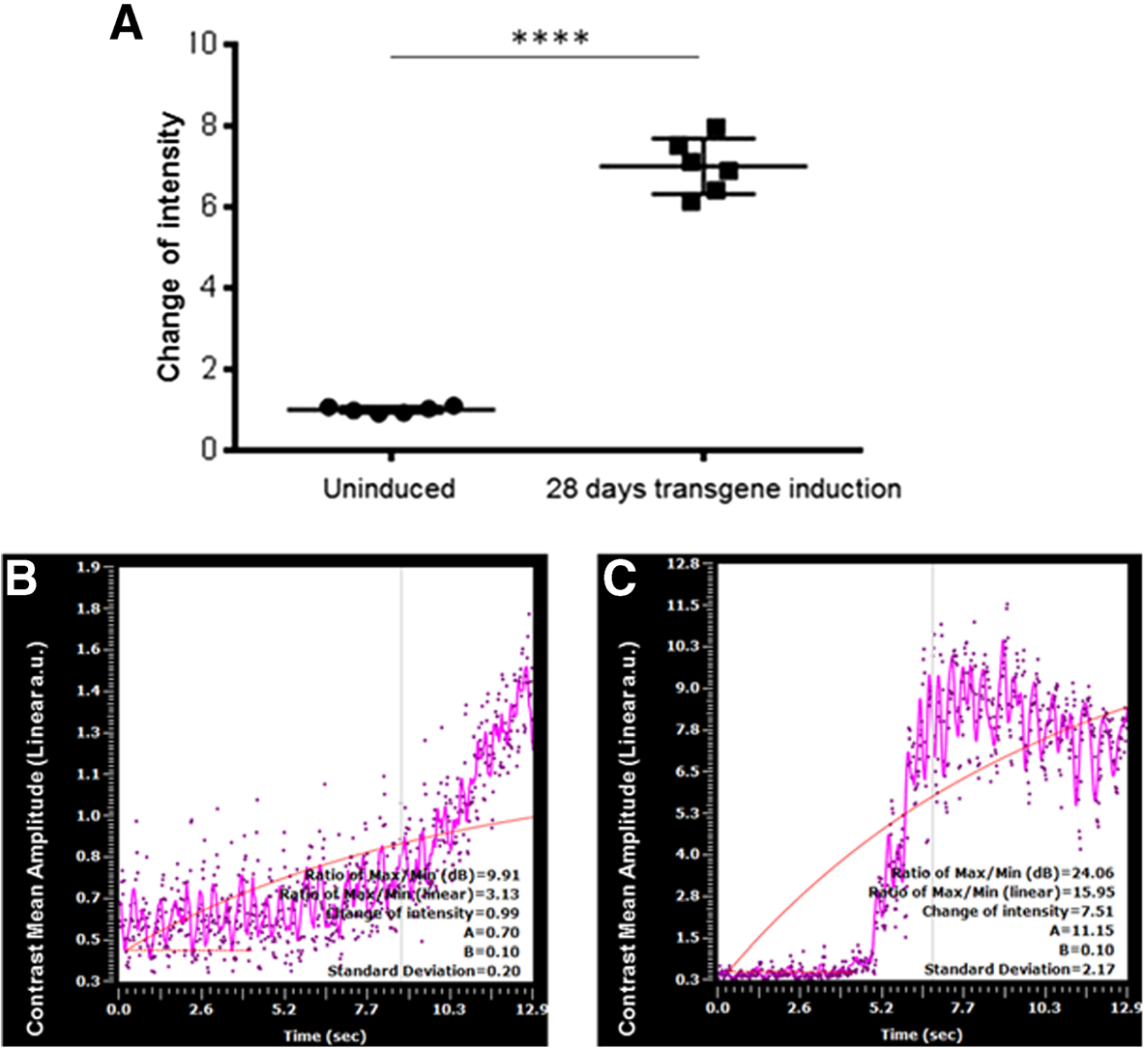
**Perfusion curve analysis of neoangiogenesis.** Eight week old mice (n=6) were left uninduced, or induced to express the stable transgene for 28 days, leading to robust neoangiogensis. **A**. Baseline (Uninduced) measurements and post-induction (28 days transgene induction) measurements of the “change in intensity” were compared, with approximately a seven-fold increase in fluorescence observed. **B**. On doxycycline (uninduced control) perfusion curve (n=6), and **C**. 28 days off dox (induced) perfusion curve (n=6). (**** denotes p<0.0001).

Perfusion curve analysis (Figure 4) confirmed the increase in functional coronary capillaries with approximately a seven-fold increase in the mean peak fluorescence intensity at day 28 of transgene induction. This result confirms that the increased coronary capillaries seen by corrosion casting are indeed functional.

### Immunohistochemistry and Western blots

**Figure 5:**
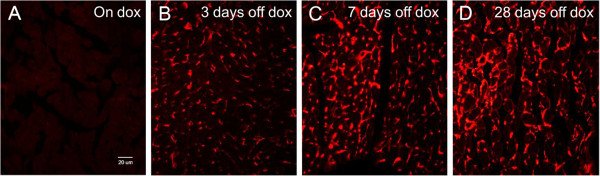
**Immunohistochemical staining of Tet-regulated transgene expressing murine hearts.** Eight week old mice were left uniduced or induced to express the stable HIF-1α-PPN-HA transgene over four weeks. **A**. HA immunohistological staining while left uninduced (On dox), **B**. HA immunological staining following doxycycline removal and transgene induction for; three days (3 days off dox), **C**. seven days (7 days off dox), and **D**. twenty-eight days (28 days off dox). A clear and progressive build-up of the stable HIF-1α-PPN-HA transgene can be seen over the course of four weeks. All images (20X); HA=hemagglutinin.

**Figure 6:**
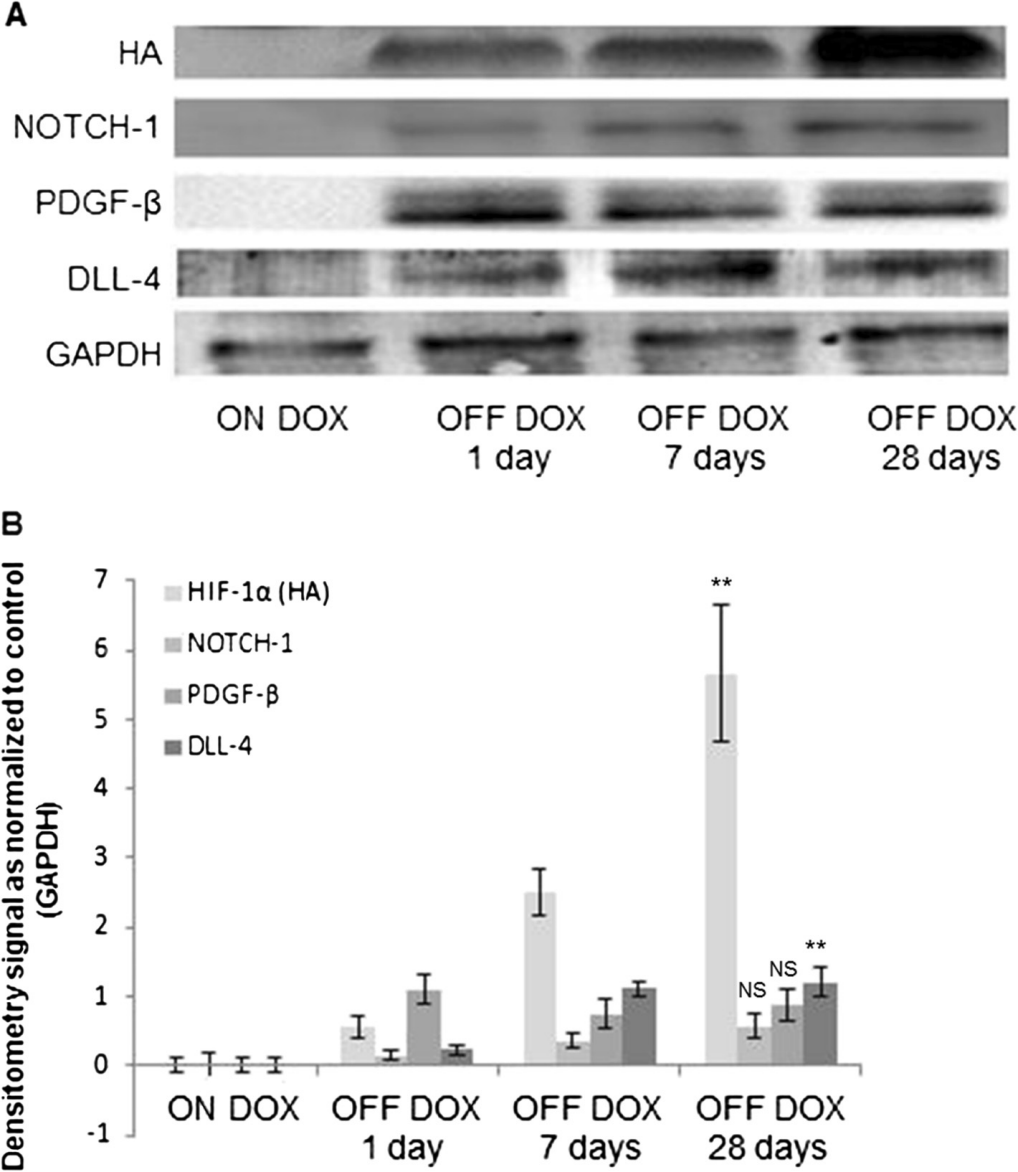
**Western blot analyses of Tet-regulated transgene expressing murine hearts. A**. Western blot images from mice left uninduced (ON DOX) and induced to express the transgene for one day (OFF DOX 1 day), seven days (OFF DOX 7 days), or twenty-eight days (OFF DOX 28 days), and **B**. corresponding densiometry analyses of the Western blot bands corresponding to; Notch-1, DLL4, PDGF-β, HA, and GAPDH. Paired *t* test analyses were conducted on OFF DOX 1 day versus OFF DOX 28 days data. (** denotes p<0.01, and NS denotes “not significant”; n=4 biological replicates for each time point).

Immunohistochemical analysis of HIF-1α-PPN-HA expression in the transgenic hearts at various time points (Figure 5) illustrated a steady accumulation of the stable transgene over 28 days, consistent with previous findings [8]. Additionally, Western blot analyses confirmed the increase in pro-angiogenic factors DLL-4, Notch-1, and PDGF-β (Figure 6), consistent with a pattern of HIF-1 induced angiogenesis [1,14,15].

## Discussion

We used a Tet-regulated, oxygen-stable, HIF transgene to create a model of enhanced HIF activity in the adult mammalian heart. Our previous experiments with this inducible model demonstrated progressive cardiac dysfunction as a result of a rapid loss of SERCA2a [8]. Taken together with our recent findings of rapid, and enhanced neoagnioegensis, it is plausible that stable HIF-1α has an angiogenesis-independent and negative effect on cardiac structure and function. In that study we also showed that although the decrement in contractility was reversible after 3 days of induction, the increase in capillary density persisted at one week after this induction, further supporting an angiogenesis-independent mechanism of cardiac dysfuction. During stable HIF-1α expression in the transgenic mice, the heart dilated and neoangiogenesis was observed. Increased capillary density was detectable after only 3 days of stable HIF-1α expression. This result is consistent with similar results following HIF-1 up-regulation [16-18]. Furthermore, this heightened vascularity persists for at least 7 days after HIF-1α expression had been terminated. This stands in contrast to increased vascularity generated by VEGF over-expression, which rapidly remits following a period of normal VEGF expression [19]. This persistent enhancement of capillary density by HIF-1 may be a result of more “mature” capillaries generated by the orchestration of several angiogenic molecules and processes. This may also be why we do not see the vascular leakage that occurs with simple VEGF expression. Our casting experiments provide a direct view of HIF-induced angiogenesis, and show that, surprisingly, the initial growth of capillaries can occur directly from relatively large vessels. We also can watch the progression of the nascent capillaries, from a mat of tightly packed small vessels into a more distributed and conventional capillary arrangement. It is encouraging that expression of a stable HIF-1 transgene produces effective angiogenesis in the adult heart, as demonstrated by the perfusion curve analysis. This supports the potential utility of this transcription factor for gene therapy [20], or other strategies for enhancing HIF activity, such as modulation of prolyl hydroxlases [21]. Previous studies have demonstrated the angiogenic potential of this molecule, but have modified HIF-1 activity during embryonic development, when vascular structures are growing and remodeling and likely to have greater plasticity. This study establishes the potential for HIF-1 to enhance capillary density after the vasculature has achieved an adult level of stability, and is supported by the temporal expression of pro-angiogenic actors in support of perfuseable neoangiogenesis.

## Conclusions

The principal findings of this study include an unusual pattern of initial capillary growth defined by casting, demonstrate functional capillary perfusion, and support the use of HIF-related strategies for therapeutic angiogenesis in adult tissues.

## Competing interests

The authors declare that they have no conflict of interests.

## Authors’ contributions

CBW contributed by: designing the experiments, performing the experiments (SEM, perfusion curve, body weight and vessel leakiness, and animal expression studies), analysing the data, and in writing and editing the manuscript. RVS contributed by: designing the experiments, analysing the data, and in writing and editing the manuscript. JO assisted with the animal experiments. JE performed immunohistological experiments and analysed data. RZA performed the perfusion study and subsequent data analyses. CDA performed immunohistological experiments and assisted in data analyses. All authors read and approved the final manuscript.
